# A Comparative Study of Enumeration Techniques for Free-Roaming Dogs in Rural Baramati, District Pune, India

**DOI:** 10.3389/fvets.2018.00104

**Published:** 2018-05-23

**Authors:** Harish Kumar Tiwari, Abi Tamim Vanak, Mark O'Dea, Jully Gogoi-Tiwari, Ian Duncan Robertson

**Affiliations:** ^1^College of Veterinary Medicine, School of Veterinary and Life Sciences, Murdoch University, Perth, WA, Australia; ^2^Ashoka Trust for Research on Ecology and Environment (ATREE), Bengaluru, India; ^3^Wellcome Trust DBT India-Alliance, Hyderabad, India; ^4^School of Life Sciences, University of KwaZulu-Natal, Durban, South Africa; ^5^School of Pharmacy and Biomedical Sciences, Curtin Health Innovation Research Institute (CHIRI), Curtin University, Perth, WA, Australia; ^6^China-Australia Joint Research and Training Center for Veterinary Epidemiology, Huazhong Agricultural University, Wuhan, China

**Keywords:** free-roaming dogs, enumeration, capture-recapture, dog counts, dog population management, rabies, mass vaccination

## Abstract

The presence of unvaccinated free-roaming dogs (FRD) amidst human settlements is a major contributor to the high incidence of rabies in countries such as India, where the disease is endemic. Estimating FRD population size is crucial to the planning and evaluation of interventions, such as mass immunisation against rabies. Enumeration techniques for FRD are resource intensive and can vary from simple direct counts to statistically complex capture-recapture techniques primarily developed for ecological studies. In this study we compared eight capture-recapture enumeration methods (Lincoln–Petersen’s index, Chapman’s correction estimate, Beck’s method, Schumacher-Eschmeyer method, Regression method, Mark-resight logit normal method, Huggin’s closed capture models and Application SuperDuplicates on-line tool) using direct count data collected from Shirsuphal village of Baramati town in Western India, to recommend a method which yields a reasonably accurate count to use for effective vaccination coverage against rabies with minimal resource inputs. A total of 263 unique dogs were sighted at least once over 6 observation occasions with no new dogs sighted on the 7th occasion. Besides this direct count, the methods that do not account for individual heterogeneity yielded population estimates in the range of 248–270, which likely underestimate the real FRD population size. Higher estimates were obtained using the Huggin’s M_h_-Jackknife (437 ± 33), Huggin’s M_th_-Chao (391 ± 26), Huggin’s M_h_-Chao (385 ± 30), models and Application “SuperDuplicates” tool (392 ± 20) and were considered more robust. When the sampling effort was reduced to only two surveys, the Application SuperDuplicates online tool gave the closest estimate of 349 ± 36, which is 74% of the estimated highest population of free-roaming dogs in Shirsuphal village. This method may thus be considered the most reliable method for estimating the FRD population with minimal inputs (two surveys conducted on consecutive days).

## 1. Introduction

Free-roaming dogs (FRD) are responsible for attacks on humans and other animals, damage to property, road accidents, contaminating the environment with faeces, spreading garbage waste and causing noise pollution (1, 2). There has been a rapid increase in the number of dogs during the last decade in India, with a concurrent increase in the number of dog bites to humans (3, 4). The large number of unrestricted, unowned, free-roaming dogs within the country is responsible for 99% of all dog bite transmitted rabies in humans (5, 6). A large uncontrolled population of free-roaming canines is also damaging to their own welfare (2, 7), as a lack of veterinary care leaves these dogs malnourished and often suffering from diseases and injuries (7, 8).

Interventions for rabies control are feasible for household pets as they generally receive adequate veterinary attention, however, such care is difficult for FRD (9, 10). The interventions usually applied to control rabies and to decrease the FRD population include culling, mass vaccination and sterilisation (10, 11). However culling does not result in a sustained reduction in the number of FRD (12, 13), and the efficacy of sterilisation on population control remains debatable (14, 15). There is a growing unanimity among researchers that mass vaccination is the best way to eradicate dog-bite related rabies (15, 16) and it is generally agreed that successful mass vaccination campaigns require 70% coverage of the dog population to achieve critical herd immunity against the disease (5, 17). However, a lack of information about the true population size of FRD raises doubts about the coverage of mass vaccination campaigns in many locations (18), and restricts critical assessment of disease intervention and population control measures and welfare issues relating to FRD (19). Although knowing the size, dynamics and demographics of the target FRD population prior to the implementation of an intervention and for post-intervention assessment is crucial (20, 21), there is no accepted standardised enumeration technique.

Formulating an enumeration methodology for FRD is very challenging not only in countries where registration and licensing of dogs is not mandatory (22), such as India, but even in countries where registration is mandatory, e.g., estimating population of free-ranging dogs in Australian indigenous communities. Various studies have used rate of capture (regression method), Beck’s method, (23), distance methods (24), extensive counts in the chosen areas and extrapolation of this number (25–27), mark-resight surveys (27, 28), Huggin’s closed capture techniques (29) and Schumacher-Eschmeyer method (30) to estimate the FRD population. There is also growing acceptance that methods for estimating the population size of wild animals yield reliable results when applied to FRD (31, 32). However, few researchers have critically evaluated and compared the different evaluation methods. As the main purpose to know the FRD population is to achieve effective vaccination coverage to eliminate rabies , rather than to accurately enumerate the population *per se*, the methods used should consider the time and monetary constraints involved, while still being reliable. In other words, while it is important to derive a robust estimate of the population size of free-roaming dogs, a method that can accurately estimate 70% of the population with minimal resource input or by using minimum number of direct count surveys is a practical requirement.

This study was undertaken in a rural setting of Shirsuphal village of Baramati Town in western India to (1) compare the estimates of the FRD population obtained with different analytical methods; (2) study the impact of extrinsic abiotic factors including temperature, humidity and wind velocity on FRD counts; and (3) recommend an enumeration technique that allows for rapid, yet robust population estimates to determine the number of FRD requiring vaccination against rabies to achieve the 70% vaccination coverage.

## 2. Materials and Methods

### 2.1. Study Area

**Figure 1:**
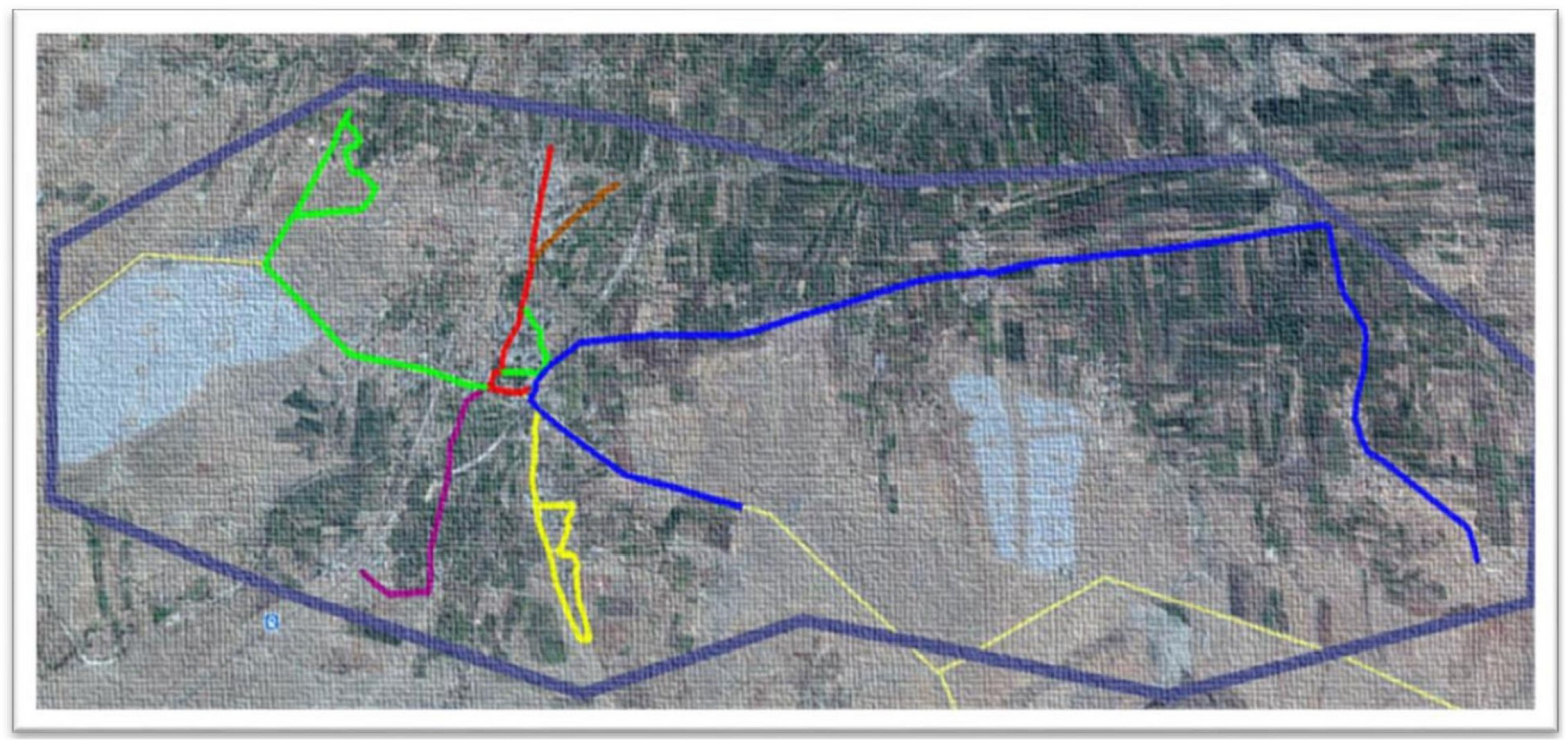
Google earth imagery (www.googleearth.com) of the village landscape and the various tracks used by the observation teams for survey (accessed on 22/07/2016). 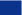 A1 (5.88 km) 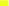 A2 (1.64 km)  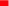 B1 (1.23 km) 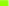 B2 (3.2 km) 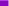 B3 (1 km) 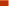 B4 (0.5 km) The light yellow lines depict the roads in areas of no human settlements. 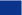 Depicts the border of Shirsuphal village.

The study was conducted in the Shirsuphal village of Baramati town located in Pune District of Maharashtra State, India in June 2016. The village comprises patches of human settlements interspersed with farmlands (Figure 1) that are connected through 16 km of roads, of which 12 are bitumen. In June the temperatures in Baramati ranges vary from 23 to 32°C, with an average humidity of 72% (https://www.timeanddate.com/weather/india/baramati).

Agriculture is the mainstay of the economy of the village with a number of poultry farms around the village that have been established over the last five years. The major land cover categories consist of agricultural fields, grazing land and protected reserve forests. No prior dog population control campaign had been undertaken in the sampled area (personal communication with village administrative head).

### 2.2. Field Methodology

Beck's definition of an FRD, “Any dog observed without human supervision on public property or on private property with immediate unrestrained access to public property” (1), was used in this investigation (33). Any dogs that were restrained or confined were excluded from the study.

The study was conducted from June 5th to June 13th, 2016 with surveys undertaken during the early mornings and late afternoons of alternate days. As photography was used to identify dogs, to ensure adequate light the surveys were conducted between 7 and 9 am and 4 to 6 pm. No surveys were conducted on the 10th and 11th June due to heavy rainfall. Surveys alternated between mornings and afternoons on five consecutive days (5– 9th June) and again on the 12th and 13th June.

Two teams of two individuals each were trained to carry out the surveys on motorcycles. They were assigned separate predetermined routes covering all the human settlements in the village. Team A rode a track of 7.52 km divided into two sub-tracks (A1 and A2) while Team B covered 5.93 km on four sub–tracks (B1, B2, B3 and B4) (Figure 1). The rider was trained to take a photograph and record the GPS waypoints while the pillion passenger completed the data sheets to record various characteristics of the encountered dog and its corresponding camera picture number. The individuals, their duties and the route ridden remained the same throughout the study. The survey was ceased subsequent to no new dogs sighted.

Each team was equipped with a motorcycle, a Garmin eTrex20 GPS device (www.garmin.com), a digital camera and a clipboard with datasheets and writing materials. Both teams started the surveys at exactly the same time on each sampling occasion and travelled on the pre-determined tracks at a speed of ~20 km/h. During the counting sessions, teams attempted not to disturb the natural behaviour of dogs by not driving too close to the animals while still maintaining their pre-set route. The teams recorded waypoints and the sex (male/female/not verifiable), age (pup/young/adult/old), size (small/medium/large), coat pattern (solid/bicoloured, tricoloured/mixed), primary and secondary colours of the coat, coat condition (good/average/poor), reproductive status (lactating/pregnant/oestrus), and overall health assessment (good/average/poor, presence of lameness, dermatitis or any other disability) of observed dogs.

### 2.3. Animal Identification and Capture Histories

In order to avoid recording the same dog by both teams each counting session was followed by ruling out any double counts that may have occurred due to the movement of dogs across the tracks. Individual animal photographs were examined and tallied with the physical attributes recorded in the datasheet. Overall, the individual identity of 98.2% (617 out of total 628 sightings from both routes) of the capture events was agreed upon by the teams to be included in constructing capture histories. Each animal was given an identity number depending on the route and the date of capture. The sighting or absence of a previously or subsequently sighted dog was recorded as either 1 or 0, respectively for each session of the survey conducted.

### 2.4. Data Analysis

Data were recorded in Microsoft Excel (Microsoft Excel, 2013, Redmond, USA). Program MARK (www.phidot.org/software/mark/docs/book/) was used to estimate the population size of the closed population using Huggin’s heterogeneity models. The “appropriate” option was selected from the Program CAPTURE option to select the suitable estimator (31, 34). The same software was also used to estimate the population size using a Mark-resight logit normal model (35). Regression analyses, Pearson’s correlation tests and χ^2^ tests were performed in R (36).

### 2.5. Population Estimation Methods

Eight capture-recapture probability techniques were used to estimate the FRD population in this study. In addition, a direct cumulative visual count of all dogs encountered during all sessions was the naïve estimate or direct count. A multiple linear regression analysis was performed to examine the effect of temperature, humidity and wind velocity at the time of the surveys on the number of sightings.

### 2.5.1. Capture-Recapture (C-R) Techniques

All the methods used in this study used the capture-recapture technique where the animals were not marked but were photographed and matched with the photographs taken on other sampling days. Most of the basic assumptions of C-R techniques, such as the interval period between surveys and complete mixing of the surveyed population, were met (23, 31). Further, the data generated in this study were subjected to test of equal catchability and test of closure (37 Page 76–83). Equal catchability of animals was assessed by calculating the G-statistic from the observed and expected number sighted during the sampling period and comparing this with the critical value of a Chi-squared distribution and a two-tailed ranked correlation test between the percentage of FRD re-sighted and the sequence of sampling occasions was used to assess if there was any trend in the numbers sighted across sessions (38). Leslie’s test for equal catchability was used to calculate the expected variance (σ^2^) and the Chi-square value (χ^2^) to check for the probability of occurrence at *p* < 0.05 for FRD known to be in the population during the survey period (37, 39). The testing for the closure of the population was based on the logic that the proportion of animals re-sighted on successive occasions would decline if a population was not closed. The Spearman’s rank correlation with the null hypothesis (H_0_ ) that, such a decline occurs in the observed data set was used to test for the closure of the population (38). The following paragraphs describe the methods followed in this study.

### 2.5.2. Regression Method

The regression line obtained by plotting the number of dogs captured on each survey session against the total distinct dogs captured until that session yields the estimate.(23, 27, 38). The catchability/detectability (*k*) on each occasion is taken as the absolute value of the intercept (b).

### 2.5.3. Lincoln-Petersen Index and Chapman’s Correction Method

The Lincoln-Petersen Index and Chapman’s correction capture-recapture methodology estimate the population size based on the principle that the proportion of animals resighted in a subsequent sample are a proportion of the marked population as a whole (27, 38, 40). Chapman (41) correction was applied to remove the bias resulting from using the Lincoln-Petersen’s estimate (27). Six estimates were obtained each for the Lincoln-Petersen Index (E_LP_) and Chapman’s correction (E_C_) from each successive pair of sightings and re-sightings. To study the temporal variation, a set of two estimates for morning surveys and three for late afternoon surveys were calculated. The two methods were then compared using a two-sample independent *t*-test.

### 2.5.4. Beck’s Method

Beck’s method (1) is an extension of the Lincoln-Petersen’s approach to multiple captures which takes into account successive recaptures following an initial effort (42). The estimate is obtained by dividing the summation of the product of total sighted and the cumulative total marked animals at large by the total number of resighted on each occasion (23, 43).

### 2.5.5. Schumacher - Eschmeyer Method

The Schumacher-Eschmeyer method states that if the total number of marked individuals is plotted against the proportion of marked samples in the t^th^ sample, the graph should be a straight line passing through the origin (*x* = 0, *y* = 0) with a slope of 1÷N, where N is the total population (44). A failure in linearity of the plotted lines implies that one or more assumptions of the closed capture method have been violated. However, if fulfilled, the N can be estimated using linear regression techniques (30, 37).

### 2.5.6. Mark – Resight Logit Normal Method

The mark-resight method takes into account the individuals that remain undetected due to individual heterogeneity and thus constitute slightly different data than for traditional methods of mark-recapture (35). Amongst the various models available, the logit-normal mark-resight estimator for individually identifiable animals with replacement was used (35 page 18–8). This method is suitable for FRD as marks are individually identifiable and the number of individuals of the primary subset are known and sighting is done with replacement (28). The Program MARK software with logit—normal estimator was used with models derived from a combination of available or fixed parameters. Time constant models with and without individual heterogeneity (*p* = p_ij_ σ_ij_=σ N(t) and *p* = p_ij_ σ_ij_=0 N(t)) were run. The sin link function was used for all model runs. The model yielding the smallest Akaike’s Information Criteria (AIC) was chosen from the available model-run options to obtain the estimate (27, 28, 35).

### 2.5.7. the Closed Capture Huggin’s Heterogeneity Model

The encounter histories were analysed using the feature CAPTURE available within the Program MARK software. The sampling design approach was similar to Horvitz-Thompson’s model as individual dogs have an unequal probability of being resighted (31, 45). The software allows the use of Huggin’s p (initial capture probability) and c (recapture probability) data type to obtain heterogeneity models which are then read with a suitable estimator (34). The “appropriate” option was selected to obtain the most suitable estimator for the population size (45–48). However, in order to fulfil the aim of the study of drawing comparisons between estimates the Program CAPTURE was run with all possible model-estimators (M_h_-Jackknife, M_h_-Chao, M_th_-Chao and M_0_), besides the one recommended by the program.

### 2.5.8. Application SuperDuplicates (AS)

The Application SuperDuplicates (AS) is a tool derived from the formula developed by Alan Turing and his colleague I.J.Good that the number of uniques/singletons (individuals that appear only once during the whole sampling exercise) holds all the information required about the undetected individuals and it was adapted by Chao et al. (49) to assess the species richness in a given area. It is also based on probability of an individuals’ resighting and we extended this technique to enumerate the population size of FRD. Each individual dog sighted at least once was counted as a unique species (Sobs – species observed). The tool utilises two kinds of data to estimate the population size: “incidence data” which are a record of the presence or absence of each observed individual in repeated samples (count and frequency), and “abundance data” which are a record of an individual observed in a single sample (50). Both abundance data and the incidence data can be used to estimate the population size. In abundance data nomenclature the individual observed in only one sampling unit is called a *singleton*, one that is seen in exactly two sampling units is called a *doubleton* and an individual seen in more than two sampling units is called a *super-doubleton.* The corresponding terms for the incidence data are *unique*,* duplicates* and* super-duplicates.* The inputs required for the abundance data are the total number of individual observations (Sobs); and the number of singletons (f1). The corresponding input requirements for incidence data are the total number of individual observations (Sobs); the number of uniques (Q1); and the number of sampling units conducted. The input was entered into the online tool https://chao.shinyapps.io/SuperDuplicates/ to estimate the population size and the percentage and number of undetected individuals (49).

### 2.6. Ethical Approval

Ethics approval for this study was granted by ATREE (Ashoka Trust for Research in Ecology and the Environment) Animal Ethics committee (AAEC) via their approval letter number AAEC/101/2016.

## 3. Results

### 3.1. Sighting Variability Between Sessions

A total of 617 reliable sightings of FRD consisting of 263 unique dogs were recorded during seven surveys undertaken over the nine-day study period. The number of unique FRD reached saturation on the 6th session with no new dogs sighted on the 7th session. The lowest count (52) was observed on the last day of the study (7th session). Wind velocity during the time of the survey had a strong negative correlation (r = −0.92, *p* < 0.01) with the number of dogs sighted in a counting session. Other meteorological variables, including temperature at the time of survey (r = −0.07, *p* = 0.39) and humidity (r = +0.07, *p* = 0.42), did not have a significant impact on the count, irrespective of the time of the survey (Table 1).

**Table 1:** Details of climatic characteristics and the number of free-roaming dogs sighted at each survey session.

Date	Time of count	Temperature (*C)	Humidity (%)	Wind velocity (Km/h)	Weather condition	Total number of dogs sighted
5/06/2016	Evening	32	55	7	Sunny	93
6/06/2016	Morning	26	80	2	Overcast	106
7/06/2016	Evening	32	55	6	Overcast	103
8/06/2016	Morning	27	78	6	Overcast	91
9/06/2016	Evening	35	42	4	Passing clouds	90
12/06/2016	Evening	30	59	13	Passing clouds	82
13/06/2016	Morning	30	70	19	Passing clouds	52

(source: http://www.timeanddate.com/weather/india/baramati).

### 3.2. Test for Equal Catchability and Closure of Population

The catchability rate was not the same across the survey period as the G statistic value (24) was significantly higher than the critical χ^2^ value of 12.6 (*p* = 0.0013). The data also failed the two-tailed Spearman's rank correlation test for equal catchability as the r_critical_ value (0.886) was less than the Spearman’s rank coefficient (0.48; *p* = 0.32); and Leslie’s test was significant (*p* = 0.0005). (37) However, the population was verified to be closed as the data passed the test for the closure of population (Spearman's rank correlation coefficient, r = 0.35 was smaller than r_critical_ value for a one-tail test ( 0.829, *p* = 0.44).

### 3.3. Regression Method

**Figure 2:**
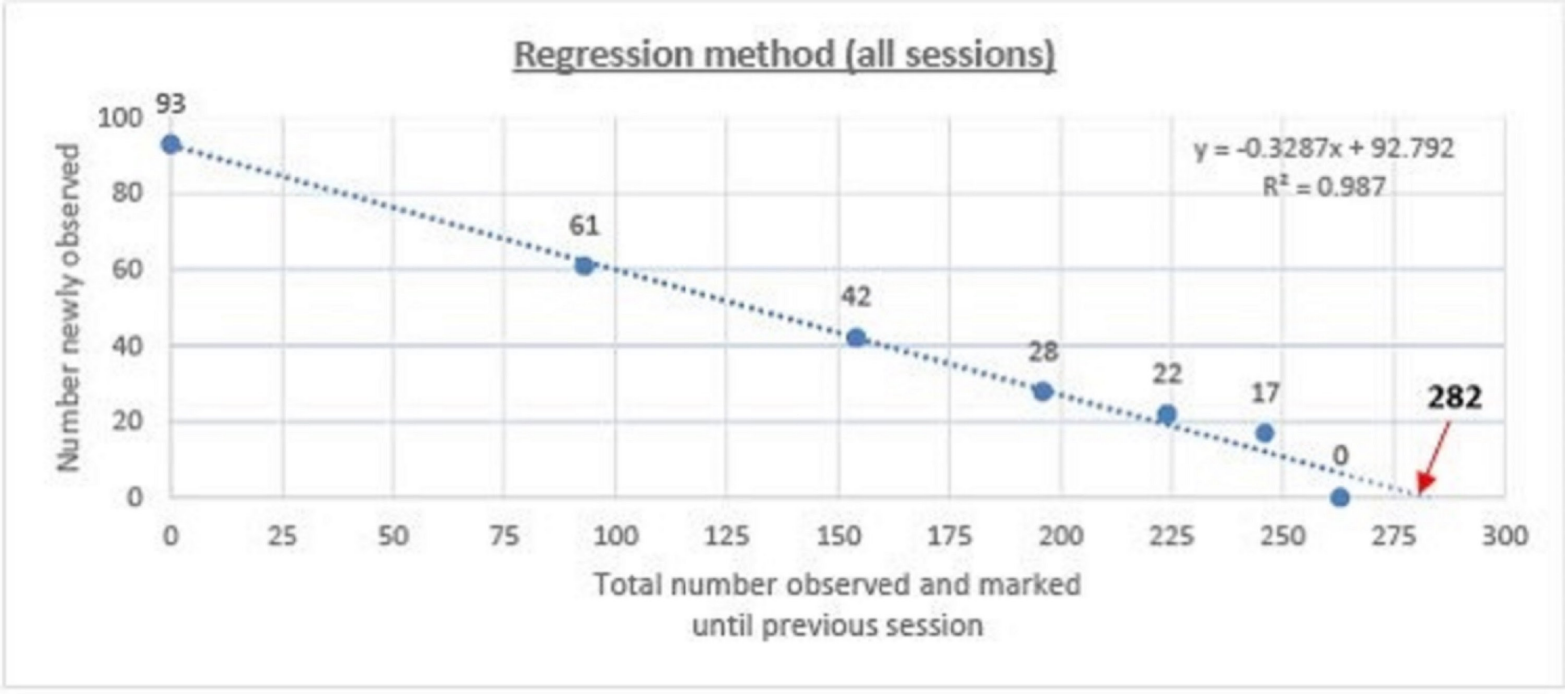
Prediction of population size of free roaming dogs by regression method for all sessions

The dog population was estimated to be 282 (95%CI 265–304, *p* < 0.001) using the regression method (*y* = −0.3287*x* +92.792, R^2^ = 0.987*)*. The overall detection probability *(P)* was found to be 0.33. The estimate when only the morning data were used was 267 (95%CI 200–1752, *p* = 0.04, *p* = 0.36) compared with 278 (95%CI 274–323, *p* < 0.001, *p* = 0.31) when only the afternoon data were used (Figure 2).

### 3.4. Lincoln-Petersen Index (L-P) and Chapman’s Corrected (C) Estimator

The estimates by the Lincoln–Petersen’s index (E_L-P_) and Chapman’s correction estimator (E_C_) for each session are shown in Table 2. The Pearson’s correlation test between the capture probability (P) for each set of counts and the estimate demonstrated a non-significant, weak negative correlation (r = −0.27, *p* = 0.6). The estimates from the two techniques were similar (two-sample *t*-test, *t* = 0.18, *p* = 0.85).

**Table 2:** Size of the free roaming dog population estimated by the Lincoln–Petersen index (E_LP_) and Chapman’s correction (E_C_) with counts on successive days.

	Days 1 and 2	Days 2 and 3	Days 3 and 4	Days 4 and 5	Days 5 and 6	Days 6 and 7	µ*
	All surveys						
E_LP_ (95% CI)	219 (184–254)	254 (209–299)	247 (199–294)	248 (194–302)	194 (160–229)	124 (106–144)	µ = 215 ± 49
E_C_ (95% CI)	214 (180–247)	248 (205–291)	241 (195–286)	242 (190–293)	189 (156–222)	121 (102–139)	µ = 209 ± 49
	^*†*^*p* = 0.48	^*†*^*p = *0.41	^*†*^*p = 0.37*	^*†*^*p* = 0.36	^*†*^*p* = 0.42	^*†*^*p* = 0.41	

	Late afternoon surveys				Morning surveys		
	Days 1 and 3	Days 3 and 5	Days 5 and 6		Days 2 and 4	Days 4 and 7	
E_LP_ (95% CI)	234 (192–275)	211 (177–244)	194 (160–229)	µ = 186 ± 54	224 (192–262)	148 (122–173)	µ = 246 ± 20
E_C_ ( 95% CI)	228 (188–268)	205 (173–238)	189 (156–222)	µ = 181 ± 54	219 (182–255)	142 (118–167)	µ = 207 ± 20
	^*†*^*p* = 0.44	^*†*^*p* = 0.43	^*†*^*p* = 0.42		^*†*^*p* = 0.41	^*†*^*p* = 0.35	

*µ is the mean of the estimates.

^†^p is the re-sighting probability of each session which is exactly the same for E_C_ and E_LP_.

### 3.5. Beck’s Method (Schnabel’s Multi-Capture Method)

The Beck’s estimate of the population was 276 (95%CI 244–317) when all 6 multiple resighting sessions subsequent to the initial sighting session were used. When influence of the temporal factor on sampling during a fixed time of the day was assessed, morning surveys resulted in a population estimate of 259 (95%CI 193–392), compared with 290 (95%CI 236–375) from the afternoon sessions (*p* = 0.67).

Table 2 Size of the free-roaming dog population estimated by the Lincoln–Petersen index (E_LP_) and Chapman’s correction (E_C_) with counts on successive days.

### 3.6. Schumacher-Eschmeyer Method

The test for the Schumacher-Eschmeyer’s method demonstrated data validity as the line obtained by plotting the number of marked (M_t_) dogs to the ratio of the total dogs sighted: resighted (C_t_/R_t_) was linear (*y* = 0.0036*x*, R^2^ = 0.9623) and passed through the intercept. The population size was estimated at 270 (95% CI 235–317).

### 3.7. Logit-Normal Mark-Resight Method

The logit-normal mark-resight method was applied with one primary and six secondary sessions. The 93 dogs sighted and photographed on the 1st day were taken as the initially marked and identified individuals. During the subsequent 6 secondary sampling sessions 61, 59, 50, 50, 49 and 20 individuals were counted as unmarked but sighted (total = 289), with 45, 44, 41, 40, 33 and 32 marked individuals sighted on each session, respectively. The estimates along with Akaike Information Criteria (AIC) scores and the mean overall sighting probability (µ) for both models are summarised in Table 3.

**Table 3:** Comparison of the models run using the Logit-normal mark-resight method on the basis of the Akaike Information Criteria (AIC).

Parameters	Model used	AIC score*	*N* ± SE.E.(95% CI)	µ
N, µ	Time constant with heterogeneity[ pij = p, σij = σ, N(t)]	936.6	334 ± 18 (307–379)	0.16
N, µ	Time constant without heterogeneity[ pij = p, σij = 0, N(t)]	1336.9	334 ± 9 (318–354)	0.16
N, µ	Time constant without heterogeneity and with fix capture probability [pij = p = 0, σij = σ = 0, N(t)]	110486.29	326 ± 89 (271–755)	–

*AIC score for Time constant model with heterogeneity is smaller and hence this represents the best model. µ is the overall mean sighting probability across the primary session and it remains the same even when heterogeneity is fixed. When capture probability is fixed to be constant for all secondary sessions and heterogeneity (σ) is assumed to absent, then the estimate (N) was a plausible value but not acceptable as the AIC score was high.

### 3.8. Huggin’s Heterogeneity Models Using Program CAPTURE

All data types selected under Huggin’s closed capture models yielded exactly the same results. No estimators and models were suggested for any of the data types and the models with the highest weights were M_th_ (0.99), M_bh_ (0.47) and M_h_ (0.40) when data from all seven days of the survey were used. The estimates after conducting the survey until saturation as derived from various estimators, along with the measure of p (capture probability) for all possible model-estimator combinations, are outlined in Table 4.

**Table 4:** Population estimates and calculated capture probability as obtained by available estimators* under Program CAPTURE.

Model	Estimator	Estimate ± SE (95% CI)	Capture probability (*p*)*^†^*
M_th_	Chao	391 ± 25.79 (350–452)	0.24, 0.27, 0.26, 0.23, 0.23, 0.21
M_h_	Jackknife	437 ± 32.57 (385–513)	0.20
M_h_	Chao	385 ± 29.83 (295–340)	0.23
M_0_	Null	283 ± 5.48 (274–295)	0.31

*M_bh_ model was not considered as behavioural variation was mitigated by photographic capture-recapture.

†Chao’s estimator for model M_th_ presents the capture probability for the 2nd to 7th session respectively.

### 3.9. Estimation Using the Good-Turing Frequency Formula Using as Tool

Estimation by Good-Turing frequency theory with *singleton* observations (abundance data) yielded a value of 392 ± 20 (95%CI 358–437) when data from all seven sessions were considered (Sobs = 263 and singletons, f1 = 118, undetected number = 129 (32.85%), duplicates = 53). The estimate for seven days of counting using the *uniques* observation (incidence data) was 375 ± 18 (95%CI 344–416) with an undetected percentage of 29.8% (112) (Table 5).

**Table 5:** Population estimates of free-roaming dogs using Application SuperDuplicates for sampling occasions ranging from 2 to 7.

Number of sample units	Number of singletons/uniques	Abundance data		Incidence data	
		Estimate ± SE (95% CI)	Undetected %(N)	Estimate ± SE (95% CI)	Undetected%(N)
7	118	392 ± 20 (358–437)	33 (129)	375 ± 18 (344–416)	30 (112)
6	123	404 ± 20 (370–450)	35 (141)	380 ± 20 (347–426)	31 (117)
5	125	390 ± 21 (354–437)	36 (144)	357 ± 18 (326–400)	31 (111)
4	121	374 ± 24 (334–428)	40 (150)	328 ± 20 (296–375)	32 (104)
3	116	354 ± 27 (309–415)	45 (158)	285 ± 18 (255–328)	31 (89)
2	109	349 ± 39 (287–441)	56 (195)	220 ± 19 (192–268)	30 (66)

## 4. Discussion

In this study we compared techniques used in ecological studies for enumeration of FRD in a rural setting, using the same operators, materials, and temporal and geographical settings to identify a robust method for estimating the population size. A reliable estimate of population size is a vital requirement for effective implementation of control measures of diseases such as rabies. A protocol that was comparable to other studies (27, 31) was used that standardised the efforts across the survey period. A direct count of the FRD, along with documentation of their characteristics, was found to be an effective and simple method to individually identify the FRD within the selected area. The counting of dogs along frequently used routes is important from resident’s point of view as these routes/roads are also locations where people are more likely to be bitten (51). In our study data collection was discontinued after no new FRD were sighted following seven days of observations conducted over nine days to enable comparisons of population estimates with different sampling efforts and methods.

### 4.1. Sighting Variations

The decline in the number of sightings over the seven survey days was influenced by climatic (rains preceding the surveys) and local factors (community event in the adjacent village) during the last two days of the survey (Table 1). Unexpectedly, Daniels (52) found that heavy rains had no noticeable effect on the behaviour of FRD. The heavy downpour in the current study could be a confounding factor as it was also accompanied by strong winds that appear to reduce dog activity as shown by lower counts on days when high wind velocity was recorded. While there was no correlation between the dog count and the ambient temperature or humidity at the time of the survey, more dogs were counted on overcast days than on clear days. Others have also reported an increase in dog activity with increasing cloud cover (52, 53). The occurrence of rains and accompanying stormy weather prior to the last two surveys and a local religious community feast in the adjacent village (as informed by the village head), a day prior to the last survey may have resulted in reduced activity of dogs in the survey location, as FRD moved away. This finding highlights the need to consider forecasted weather, as well as human events, during the planning of enumeration surveys. The attentiveness of the observer has also been highlighted as a factor that affects detectability (54). Conducting more surveys may introduce fatigue amongst observers, resulting in a decrease in counts as the study progresses.

### 4.2. Capture-Recapture (C-R) Techniques

The assumption of a closed population was validated in this study as proportion of re-sightings across the sessions did not vary significantly. This confirmed the suitability of C-R techniques for estimating the FRD population due to the short duration of this survey. However, the catchability rate did vary significantly across the survey sessions due to differences in the recaptures (re-sightings) arising from weather conditions and and sociological factors (organised community events) may influence the count on a particular day. Conducting counts during the mating season, when males may move greater distances, could also result in differences in counts. As the methodologies used in this study considered the resighted number without accounting for such extrinsic factors, the assumption of equal catchability across the survey sessions was not unexpectedly violated.

### 4.3. Types of C-R Estimation Techniques

#### 4.3.1. Methods Not Based on Individual Identity

The Lincoln-Petersen’s index and the Chapman’s corrected method have advantage over other methods as they require just two sighting sessions. The estimates of both N_L-P_ and N_C_ were lower than N_B_, N_S-E_ and N_R_ which is supported by the findings of a comparative study in Brazil (55). We established that the estimate is influenced by counts from each day but not by resighting probabilities (r = −0.27, *p* = 0.6), implying that the estimate may still vary irrespective of identical sighting probabilities (Table 2). The number of resights oscillates with the total sightings of the day, so that if extrinsic factors, such as weather conditions, result in a large drop in the total count, the corresponding resights will also decrease, and thus their reliability for determining the population size for adopting a mass vaccination program is questionable. Thus, we do not recommend the Lincoln-Petersen’s index or the Chapman’s corrected estimator for FRD.

In comparison, the regression method was found to be robust with a higher count (N_R_) than other multi-capture methods yielding higher precision and smaller SE than N_B_ (Table 6). This is contrary to a study by Fei et al. (23), where simulations for N_R_ and N_B_ were compared and N_B_ was found to be a better estimator as N_R_ failed to deliver reliable estimates at low capture probabilities, even when the number of survey occasions was higher. However, in this study, the N_R_ was higher than N_B_, even when the capture probability (k = 0.382) was much smaller than the mean P for Beck’s method (0.629) with the same sampling effort. Beck’s and the Schumacher-Eschmeyer’s methods resulted in a comparable estimate of N (N_B_ = 276, N_S-E_ = 270) (Table 6). This was not unexpected as both methods rely on individuals resighted on successive sessions.

**Table 6:** Comparison of the population size from direct counting with estimates obtained using 8 different capture-recapture methods until saturation (7 survey occasions spread over 9 days).

Method	Estimate ± SE (95% CI) (numbers)
Direct method	263
Lincoln-Petersen’s estimate	254 (209–299)
Chapman’s correction	248 (205–291)
Beck’s method	276 (244–317)
Schumacher-Eschmeyer’s estimate	270 (236–317)
Regression method	282 ± 94 (265–304)
Log-Normal Mark Re-sight method	326 ± 15 (303–364)
Huggin’s methods	
Model M_th_ (Chao estimator)	391 ± 26 (350–452)
Model M_h_ (Jackknife estimator)	437 ± 33 (385–513)
Model M_h_ (Chao estimator)	385 ± 30 (340–458)
Good- Turing (Application SuperDuplicates)	380 ± 19 (347–426)

The Schumacher-Eschmeyer’s method applied by Totton et al. (30) to study the effect of sterilisation on the population size of FRD in Jodhpur, India, emphasised the graphical test to ensure non-violation of assumptions without describing them. Assuming similar assumptions as for closed populations, the straight line plotted signifies the relationship between the fractions of identified animals on each sampling to the total number of animals identified prior to that sampling and the slope of the line of best fit gives N_S-E_. A practical aspect of capture-recapture in FRD, especially in countries with large dog populations such as India, is that there would seldom be a sampling occasion with no or a very small fraction of re-sightings. As a consequence, the line of best fit terminates sooner (when y = 0, or when saturation is reached) as compared to other species (e.g., fish) where the point of saturation is difficult to meet. The inappropriateness of the Schumacher-Eschmeyer method was highlighted by Belo et al. (32) who considered this method was more appropriate for aquatic species rather than terrestrial ones, especially dogs. Beck’s method has been used for estimation of feral dog populations in Brazil, Mexico and India (31, 32, 53). As Beck’s estimator also relies on the ratio of marked animals sighted on the sampling day to the cumulative number of animals sighted before the start of that sampling, predictability is limited by a strong plausibility of reaching saturation. Thus, both these methods tend to underestimate the population size of FRD as dogs are not difficult to resight in rabies endemic areas. This is also supported by the finding that the estimates using this method are similar to the total number of FRD actually sighted at least once during the survey (*N* = 263). As the primary aim of this study was to arrive at an estimate for effective vaccination coverage (70%) to keep R_0_ <1, the difference between the estimates is not high enough to instil any confidence on deciding the number for effective vaccination coverage.

####  4.3.2. Methods Using Individual Identity

We applied the Logit-normal mark-resight model as the sightings were accomplished without replacement, the intervals between surveys were small but uniform and repeat sightings of an individual during a particular sampling session were negligible. The survey design used in this study allowed only one primary sighting survey. If the survey continued long enough to start another primary, the interval may have violated the assumption of a closed population. The estimate was found to be comparatively precise (small SE) compared to the Huggin’s heterogeneity models but its accuracy may still be debatable, as only one primary was conducted. The estimate was much lower than the Huggin’s model (Table 7), thus raising concerns about it being an under-estimate.

**Table 7:** The population estimates obtained by the Huggin’s heterogeneity models compared with Application Superduplicates (AS) online tool based on Good-Turing frequency formula on successive reduction of sampling efforts.

ESTIMATES (numbers)								
Number of survey effort	M_h_-Jackknife ± SE	95% CI	M_h_-Chao ± SE	95% CI	*M_th_-Chao ± SE	95% CI	*^†^*AS ± SE	95% CI
2	207 ± 9	193–228	286 ± 34	235–371			349 ± 39	287–441
3	302 ± 15	277–335	321 ± 31	274–396	493 ± 103	347–772	354 ± 27	309–415
4	371 ± 21	336–418	352 ± 31	305–428	371 ± 34	318–455	374 ± 24	334–428
5	429 ± 28	383–492	384 ± 33	333–465	390 ± 27	343–460	390 ± 21	354–437
6	467 ± 34	410–546	400 ± 27	356–464	400 ± 33	350–480	404 ± 20	370–450
7	437 ± 33	385–513	385 ± 30	340–458	391 ± 26	350–452	392 ± 20	358–437

*The M_th_-Chao model could not project any estimates after single resight survey due to lack of temporal data.

*^†^*Application Superduplicates.

In the case of the CAPTURE feature of Program MARK, abundance is predicted by the conditional estimator model, which uses capture histories of individuals seen at least once, was selected (46, 56). This method was used by Belsare and Gompper (31) using the M_h_ model to estimate the population size of FRD in a nearby locality in India during a mass vaccination programme. Using the Jackknife estimator that study concluded that Huggin’s closed capture models yielded higher population estimates than the Beck’s estimator as the latter was found to be even lower than the number of identified dogs that were vaccinated.

In the current study, however, the feature CAPTURE could not suggest an appropriate estimator, which was likely due to the nature of the input data, e.g., the time variation is evident for the last session when the count was the lowest, but reasons were not temporal but environmental (heavy rains) and sociological (community feast). The Program CAPTURE algorithm nevertheless identified it as a temporal and/or behavioural factor as apparent from the rank and weight of the models (M_th_ = 0.99, M_bh_ = 0.47, M_h_ = 0.4). The second best weight was given to model M_bh_ indicating that model selection was influenced by the sharp drop in the count on the last day. As the behavioural variation (generally used for trap-shy or trap-happy behaviour) is more of a capturing effort attribute (34) and it is negated by using photographic capture-recapture, considering the M_bh_ model for population estimation would be misleading. The best-suggested model, M_th_ (weight = 0.99), and M_h_ (weight = 0.4), were run using Chao and Jackknife estimators, respectively. The M_h_ model with Jackknife estimator obtained a higher estimate (437 ± 33) than M_th_ with Chao (391 ± 26), however, the capture probability of M_h_ –Jackknife (*p* = 0.20) was lower than the average capture probability of M_th_-Chao (*p* = 0.24). This implies that, implying that M_h_-Jackknife overestimates the population size if nearly all animals are captured (42 page 63), which could be the case in this study where most animals were captured at least once. However, if the survey was continued after the 7th survey, it would result in increasing the number of resights without adding to the total unique individuals sighted and tend to reduce the estimate. Hence, we recommend stopping the survey, once saturation is reached.

The “abundance data” estimates from the Good – Turing formula using the Application SuperDuplicates (49) after seven sampling surveys were identical with the estimates resulting from the Huggin’s model M_th_ with estimator Chao (Table 7). The “incidence data” may appear to be a better model as it considers the number of sampling units and this study found that the duplicate estimates were closer to the actual figures than the doubleton estimates; the population size estimate, however, was less than the “abundance data”. Although the difference between the estimates may not appear substantial assuming them to indicate the true population, the difference widens with reduction of sampling efforts (Table 5) which doesn't augur well if we want to get a reliable estimate with minimal efforts (counts). This suggests that “abundance data” is a better option over “incidence data” to generate a reliable estimate of FRD.

### 4.4. Comparison of the Methods Used in This Study

The primary aim of this study was to recommend an enumeration method that could provide a reliable estimate of the population size of FRD to achieve effective vaccination coverage against rabies. The methods that do not include heterogeneity and assume equal catchability across resight surveys are not reliable estimators and thus are not recommended for FRD population size estimation, whereas the methods that consider the heterogeneity of the individuals provided comparatively robust estimates. Even among the C-R methods which include the influence of heterogeneity, the Logit-normal mark-resight method could only be run with one primary and provided an estimate lower than other C-R methods, and thus is unsuitable for FRD estimation for rabies control. Thus Huggin’s heterogeneity models and the AS tool are considered acceptable methods for estimating the FRD population. As these methods were run on data using exactly the same resource input, a comparative study of their respective estimates with diminishing resource input helps in the selection of the method that would give a reliable population estimate (Table 7).

The estimates derived from the various methods (Table 7), clearly show that the estimates decrease across all methods on the 7th survey. This is because the saturation point was reached on the 6th survey and further resights do not add any new information. While this keeps the pi (individuals not seen at all) part of the data unchanged while reducing p (seen once), the overall estimate tends towards stabilisation. It can, however, be inferred that the estimate after the 6th survey is the highest possible, albeit a likely slight overestimation.

Further examining the trends with reduced inputs (Table 7), we find the estimates decrease when data from fewer surveys are included, except for model M_th_-Chao (due to reduced temporal information available to the Program CAPTURE algorithm with two/three surveys). This helps remove M_th_ model from the choice of methods to use as our endeavour is to obtain a reliable estimate using a minimum number of surveys.

**Figure 3:**
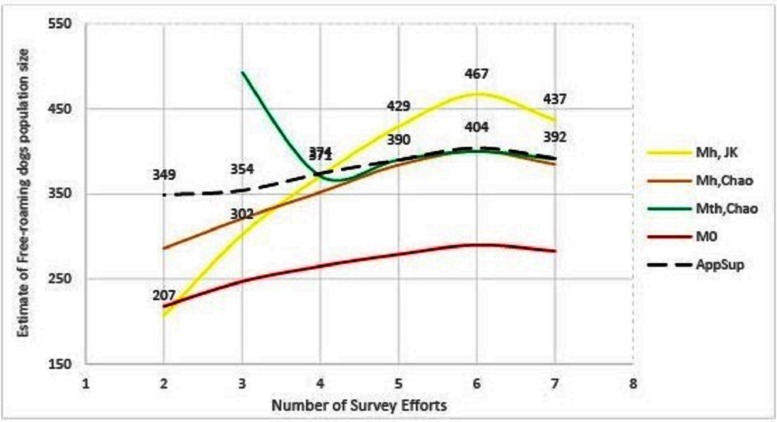
Graphical representation of the trend of population estimates using Huggin’s models and Application SuperDuplicates (AS) with the number of survey sessions. Mh, JK = model M_h_-Jackknife, Mh, Chao = model M_h_-Chao, Mth, Chao = model M_th_-Chao, M0 = model M_0_ and AppSup = Application SuperDuplicates.

The remaining methods (M_h_-Jackknife, M_h_-Chao and Application SuperDuplicates) also show diminishing estimates with a reduced number of surveys, however, there is a wide variation in the rate at which they fall (Figure 3). The steepest fall is seen by the M_h_ –Jackknife model with an estimate of 207 ± 9 compared to M_h_ –Chao (286 ± 34) and AS tool (349 ± 39) at minimum input effort. A smaller estimate is not unexpected as the CAPTURE algorithm has less data that adversely affects its accuracy. The negative bias of the model M_h_- Jackknife was explained by Chao (57) who reported that smaller sampling efforts (<5) reduced its precision. Otis et al. (58), page 34 that the M_h_-Jackknife has a tolerable bias when trapping occasions are sufficiently large (>5), indicating that the estimate by this method after six surveys (467 ± 34) may be closer to the true population. This rationale was used by Belsare and Gompper (31) to recommend M_h_-Jackknife as the most appropriate model-estimator to assess the population size of FRD in India.

The M_h_ –Chao yielded an estimate of 286 ± 34, which, although better than M_h_-Jackknife combination, is still a smaller number if we consider the M_h_-Jackknife with six surveys as approximating the true population. It has been mentioned that Chao’s estimator works well with sufficient capture occasions (usually >5) (42, 59 page 69–73). Consequently, this estimate does not provide us with a reliable estimate to achieve 70% herd immunity and thus the M_h_-Chao is also not a recommended method in this situation.

The AS tool developed by Chao et al. (49) based on Good-Turing theory, however, obtains a sufficiently large estimate (349 ± 39) with only two surveys or with one set of sight-resight data. Considering M_h_-Jackknife estimate after six surveys (467 ± 34) to be close to the true population, the “abundance data” estimate by AS tool is 74% of the true population. However, before recommending this as the best method to estimate the population size of FRD, it is pertinent that we crosscheck the reliability of estimates by the AS tool in comparison to other model-estimator combinations that are known to be accurate with sufficient (>5) sampling events. We found that the AS-tool estimate after seven surveys (392 ± 20) was similar to the M_th_-Chao (391 ± 26) and M_h_-Chao (385 ± 30), although lower than M_h_-Jackknife (437 ± 33). It is interesting to note that barring M_h_-Jackknife, other estimates are similar after six surveys as well (Table 7). However, we can infer that the AS-tool estimate, after a single set of a sight-resight exercise, is a robust estimator of at least 70% of the true population size of the FRD in Shirsuphal village. The introduction of AS tool by Chao et al. (49) based on the Good-Turing theory, fortunately, solves most of the complexities of choosing the datatypes, model and estimator selection associated with program CAPTURE and obtains a dependable number to work towards achieving 70% vaccination coverage in FRD against rabies. Nonetheless, there is an inherent shortcoming of the AS software, namely that the output generated for the SE and 95% CIs differ slightly on repeats, even when exactly the same data are entered (49); this, however, does not affect the total estimate.

The major limitations of this study are firstly the absence of a gold-standard estimate against which the estimates can be compared with and thus we had to be content with the largest estimate, which could be an overestimate of the true population. Secondly, as these techniques have been developed and perfected for wildlife enumeration, we surmise the sighting probability for FRD in human frequented habitat is influenced by factors probably not applicable to other ecological studies. Finally, we admit that the study is based on limited tracks as the inclusion of the interior tracks of the village was not possible due to limited resources and time constraints. However, we recommend extensive surveys should be carried out in the future to generate a gold-standard estimate to compare and validate the findings.

In this study, we compared most of the available enumeration methods that can be applied for estimating populations of FRD, except for the Spatially Explicit capture-recapture (SECR) and methods based on Bayesian models. The former could not be used as it requires intensive spatial preparation of the data in a GIS, and the latter needs use of reliable priors which are not present for FRD in the study location. The distance method was not used as it is more appropriate for density estimates rather than total population size and because dogs are not distributed randomly in relation to the transect lines (32). The importance of the various models of Huggin’s closed capture models cannot be overlooked for future studies where empirical priors can be evolved for population size estimations based on Bayesian models. We recommend further testing the applicability of the Application SuperDuplicates software for FRD enumeration study in different locations and conditions.

## Ethics Statement

This survey involved in this study required observation of the free-roaming dogs in Shirsuphal village of Baramati town and ethical approval for the same was granted by ATREE (Ashoka Trust for Research in Ecology and the Environment ) Animal Ethics committee (AAEC) vide their approval letter number AAEC/101/2016.

## Author contributions

All authors have contributed and approve the contents of this article. HT developed the study, collected and analysed the data. HT, IR, AV, MO and JT wrote the article, provided critical revision and helped interpretation of contents and implications.

## Conflict of Interest Statement

The authors declare that the research was conducted in the absence of any commercial or financial relationships that could be construed as a potential conflict of interest.
